# Risk Factors Before Dialysis Predominate as Mortality Predictors in Diabetic Maintenance Dialysis patients

**DOI:** 10.1038/s41598-019-46919-w

**Published:** 2019-07-23

**Authors:** Noa Tsur, Idan Menashe, Yosef S. Haviv

**Affiliations:** 1 Department of Public Health, Faculty of Health Sciences, Ben-Gurion University, Beer-Sheva, Israel; 2 Department of Nephrology, Soroka University Medical Center, Beer-Sheva, Israel

**Keywords:** Risk factors, Haemodialysis

## Abstract

Diabetic patients undergoing maintenance dialysis (MD) have a particularly high mortality rate. Many of the risk factors for mortality have been identified in diabetics who die before reaching end stage renal disease (ESRD), i.e. before dialysis (BD). In addition, many risk factors for mortality have been identified in diabetics after dialysis onset (AD). However, whether in the BD period there are long-term risk factors for AD mortality in diabetics is unknown. We therefore investigated a new concept, i.e. that clinical and biochemical risk factors during the BD stage affect long-term AD mortality. We performed a population based retrospective cohort study, in diabetic CKD patients in a single center in south Israel who initiated MD between the years 2003 and 2015. Clinical and biochemical data 12 months BD and 6 months AD were collected and evaluated for association with mortality AD using Cox’s proportional-hazards model. BD parameters that were found to be significant were adjusted for significant parameters AD, thus generating a “combined” regression model in order to isolate the contribution of BD factors on long term mortality. Six hundred and fifty two diabetic MD patients were included in the final analysis. Four independent BD parameters were found in the multivariate model to significantly predict AD mortality: age, BMI (inversely), pulse pressure (U-shaped) and cardiovascular comorbidity. AD independent risk factors for mortality were age, BMI (inversely) and albumin (inversely). Of note, BD factors remained dominantly significant even after additionally adjusting for AD factors. No association was found between either BD HbA1C levels or BD proteinuria and AD mortality. In diabetics who reach ESRD, BD parameters can predict long term AD mortality. Thus, some of the factors affecting the poor survival of diabetic MD patients appear to begin already in the BD period.

## Introduction

CKD in general and ESRD in particular confer a high risk for cardiovascular mortality, further enhanced by diabetes^1^. Despite improvements in dialysis technique, mortality remains high. Life expectancy for patients in maintenance dialysis (MD), according to the USRDS report is 4.5 years for patients aged 60 to 64, a time expectancy that is shorter than for most of the malignancies. Diabetic MD patients have a 1.3-fold higher mortality rate relative to other primary renal disease^2^.

Several risk factors predicting mortality after dialysis onset (AD), have been described including albumin levels, BMI below 30, vascular access, anemia, inflammation and comorbidities including a history of cardiovascular disease^3–7^.

In 2009 Lamiere *et al*.^8^ had introduced the hypothesis that the dismal AD survival of the general MD population derives from BD cardiovascular factors. In the current study we focus on diabetic MD patients and evaluate BD vs. AD risk factors predicting AD mortality.

Several studies had presented the phenomenon of “long-term effect” of certain risk factors, where strict adherence to controlling early risk factors such as hypertension and hyperglycemia may have a long-term prognostic effect on mortality years after the research period of tight control. This principle of long-term benefit was demonstrated for intensive blood pressure control among patients with CKD in the extended follow up of the MDRD trial^9^, patients with diabetes and cardiovascular risk factors in the follow-up of the ACCORD study^10^, and among patients with cardiovascular risk factors in the ADVANCE-study-follow up^11^. In another study^12^, the phenomenon was demonstrated in patients with type 1 diabetes with regard to glycemic control. In contrast, the follow-up study of the UKPDS trial^13^ has not found a long-term benefit for tight blood pressure control in diabetic patients.

Most studies predicting AD mortality included AD factors rather than BD factors. Thus, to the best of our knowledge the only BD risk factors shown to predict AD mortality were comorbidities such as a history of cardiovascular disease or cancer, diabetes mellitus as the primary kidney disease (3), blood pressure^9^, and HbA1C^14^. In diabetic patients HbA1C levels 6 months BD either showed a strong association with mortality and cardiovascular outcomes AD (14) or not^15^. In a mostly non-diabetic population the long-term follow up of the MDRD study^9^ showed that although strict blood pressure control during CKD did not prevent progression to ESRD, it had a beneficial effect on mortality AD.

Thus, it appears that BD risk factors for AD mortality are less established in general and in diabetic MD patients in particular. We therefore asked what BD risk factors in MD diabetic patients may affect AD mortality. To this end we analyzed BD and AD risk factors and compared their association with AD mortality.

## Methods

### Study design and population

We performed a population based retrospective cohort study of members of Clalit Health Services (CHS), the largest medical service provider in Israel, who began MD from January 1, 2003 to December 31, 2015 and had a prior diagnosis of diabetes. The study was approved by CHS Soroka hospital IRB and all data collection methods were performed in accordance with the relevant guidelines. As this was a retrospective study where the data was de-identified, it was exempt by the IRB from the need for informed consent.

Patients were excluded if their dialysis treatment period or total follow-up time were shorter than 90 days. Patients were followed up until renal transplantation, death, or 31 December 2016. To improve accuracy, data were collected starting from 2002, when computerized registration began.

To include only MD patients with a high probability of diabetic nephropathy, patients with other known primary renal disease or those who developed diabetes only AD or patients that started MD prior to the age of 40, were excluded.

### Clinical definitions and data sources

We identified patients with evidence of MD treatment based on the International Classification of Diseases, 9th revision (ICD-9) codes. Data on ICD-9 diagnoses in the hospital and in community clinics, demographic data and laboratory tests results were captured from the CHS computerized database.

Diagnosis of diabetes mellitus was defined by either a laboratory test with fasting glucose ≥126 mg/dl in two different tests, hemoglobin A1C ≥ 7%, a diagnosis of diabetes in the hospital or community records or by the use of a hypoglycemic agent.

For each patient undergoing MD between 2003 and 2015, clinical and biochemical data were collected 12 months BD and 6 months AD. Data on comorbidity and mortality were collected as well until study end.

### Statistical analysis

Univariate Cox regression models were used to test the effect of demographic and clinical parameters on patient’s long-term mortality. Variables were examined continuously and categorically to check for their optimal form based on model quality indices. Proportional hazards assumption for each variable was assessed and validated. Variables with significant effect at the significance level of PV < 0.1 were further included in the multivariate analyses. Possible correlations between parameters with PV < 0.1 were tested, and in case of high (r > 0.7) significant co-linearity, the more significant parameter of the two was inserted into the multivariate model.

Multivariate Cox regression models were constructed on the same follow-up time for variables in different time periods: Model-1 included clinical variables collected six months AD; Model-2 included clinical variables collected one year BD; Models 3A, 3B and 3C integrated together BD and AD parameters that were significant in the univariate models in order to evaluate an independent long-term effect of each variable. We used forward-LR stepwise regression procedure to select variables for each of these models using P < 0.05 for variable entrance, and P < 0.1 for variable removal.

### Definitions of the research variables

#### Dependent variables

Mortality - survival rate: calculated according to the time elapsed from MD initiation to mortality or until the date of transplantation or until the end of the study on 31.12.2016. An event was defined as death from all causes (“all-cause mortality”).

Independent variables: The demographic and clinical baseline variables were taken at initiation of MD (time 0). The biochemical variables one-year BD were taken from the results of the last test that was performed at least one-year BD. The biochemical variables AD were taken according to the results of the tests that were performed closest to 6 months AD.

Parameters were collected and referred to as follows: Age, triglycerides, albumin, HDL, LDL, cholesterol and phosphorus were presented as continuous variables. Socioeconomic status (low, Intermediate, high) was defined by CHS categorization according to Zip code of community clinic. Smoking status (never smoked = 0, ever smoker = 1) and comorbidities were categorized as yes or no. Comorbidities “BD” and “AD” were taken from the hospital and community records according to ICD 9 in all period before/after starting MD for the following comorbidities: heart failure, CVA, coronary heart disease (CAD), myocardial infarction (MI), peripheral vascular disease (PVD), chronic obstructive pulmonary disease (COPD), and heart failure (HF). Comorbidities were grouped to “cardiovascular-comorbidities” (MI,CVA,CAD,PVD,HF) and “vascular comorbidities”-(without HF). Diabetes duration was categorized as <10 or >10 years. Date of Diabetes onset was determined as the earlier date of diagnosis. Type of renal replacement therapy was categorized by first MD initiated: hemodialysis, peritoneal dialysis or transplant BD. Albuminuria, hemoglobin, Hba1c, BMI, were analyzed as continuous and categorical variables to validate best presentation. Categorical groups were as follows: Body mass index (BMI) was calculated from the recorded height and weight and stratified into 5 categories according to the WHO definition^16^: normal -BMI 18.5–24.9, overweight 25–29.9, obese class 1 (30–34.9), obese class 2 (35–39.9), obese class 3 (≥40).

To avoid bias, patients with BMI < 18.5 were excluded because of the known higher mortality risk in this group^17^. HbA1c was divided as < = 7% (reference) and >7%^18^. Hemoglobin was divided into 3 groups: <10,10–13, >13, with 10–13 being ref.^19^. Albuminuria and proteinuria were diagnosed by at least 2 of 3 positive tests a year BD by either albumin to creatinine ratio (ACR) mg/g, or by 24 h urine for albumin collection (mg/L) and divided into 3 groups: normoalbuminuria = ACR < 30 mg/g or <30 mg/L, microalbuminuria 30–300 mg/g or 30–300 mg/L, macroalbuminuria = >300 mg/g or >300 mg/L. Blood pressure (BP) was divided into 4 groups according to the WHO categorization: systolic BP (SBP) < 120 (reference), 120–139.9, 140–159.9, >160. Diastolic BP (DBP) < 80 (reference), 80–89, 90–99, >100. Pulse pressure (PP) was defined as the difference between the SBP and DBP, classified to 3: PP < 40, 40–60 (being reference), PP > 60. In MD blood pressure measurements were collected from clinic or hospital data and not from BP measurements taken during dialysis. eGFR was calculated according to serum creatinine test results one year prior to dialysis using the MDRD equation^20,21^.

## Results

**Figure 1:**
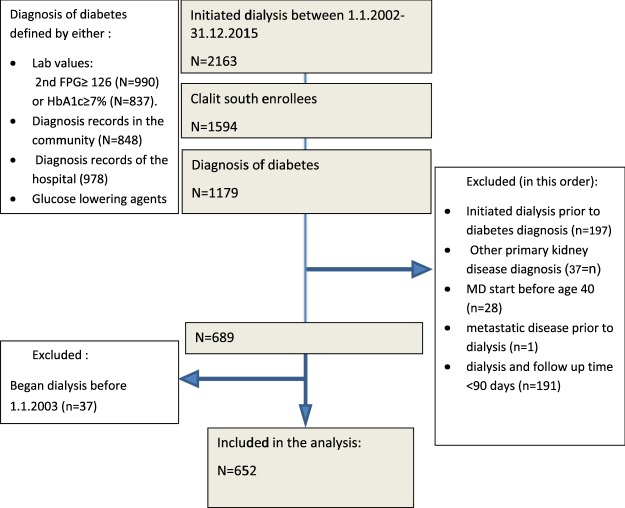
Flowchart describing patient inclusion and exclusion criteria.

Included were 652 diabetic MD patients (Fig. 1). The mean follow-up period was 6.73 years, with a range of 1 to 13 years. Of these, 431 patients (66%) died during the follow-up time. This resulted in a mean and median survival times of 1655 ± 60 days (~4.5 years) and 1323 ± 66 days (~3.6 years) respectively.

Baseline demographic and clinical characteristics at initiation of MD and their effect on mortality are presented in Table 1. Average age was 67.6 with 60% having a major comorbidity and 98% diagnosed with HTN.

**Table 1:** Demographic and clinical characteristics of patients with diabetes at initiation of MD and the associated HR on AD mortality*.

	N (%) or Mean ± SD	B	p-value	HR (95% C.I)
Age at ESRD onset (years)	67.6 ± 9.37	0.034	<0.001	1.035 (1.025–1.045)
Gender Male	372 (57.1)	0.046	0.638	1.047 (0.86–1.26)
Race (Bedouin)	76 (11.7)	−0.169	0.304	0.844 (0.612–1.165)
Socioeconomic status^a^ -low	490 (75.3)	[Reference]		
Socioeconomic status - intermediate	147 (22.6)	0.258	0.024	1.294(1.034–1.6)
Socioeconomic status -High	14 (2.2)	−0.078	0.817	0.925 (1.034–1.6)
Diabetes duration until ESRD onset	9.47 ± 4.4	0.025	0.042	1.025 (1.001–1.050)
Diabetes duration >10Y	305 (46.8)	0.21	0.047	1.22 (1.02–1.49)
Smoker ever	261 (40)	0.05	0.607	1.052 (0.868–1.275)
Comorbidities^b^				
Hypertension	640 (98)	−0.259	0.25	0.77 (0.49–1.2)
CAD-BD	342 (52)	0.198	0.041	1.21(1.008–1.47)
COPD-BD	28 (4)	0.285	0.285	1.33(0.84–2.09)
HF-BD	247 (38)	0.428	<0.001	1.53(1.26–1.86)
MI-BD	192 (29)	0.315	0.003	1.37(1.11–1.68)
Stroke –BD	62(9.5)	0.403	0.013	1.496(1.088–2.05)
PVD- BD	103(16)	0.389	0.002	1.47 (1.15–1.88)
Vascular Comorbidity^c^ BD	385 (59)	0.289	0.003	1.33 (1.098–1.62)
Cardiovascular Comorbidity^d^ BD	422 (64.7)	0.399	<0.001	1.491 (1.2–1.83)

*Cox univariate survival analysis.

^a^Socioeconomic status defined by home clinic according to “Clalit-health-service” categorization.

^b^All the comorbidities were diagnosed prior to the initiation of dialysis.

^*c*^Including: MI, CAD, PVD, Stroke.

^*d*^Including: HF, CAD, MI, PVD, stroke.

Abbreviations: SD: Standard deviation, HR: hazard ratio, CI: confidence interval, ESRD: end stage renal disease; BD: before dialysis, HF: heart failure, CAD: coronary artery disease, MI: myocardial infraction, PVD:peripheral vascular disease.

### Demographic and clinical risk factors

Older age, diabetes duration >10 years, intermediate vs. low socioeconomic status, heart failure (HF) and BD cardiovascular comorbidity were significantly associated with higher mortality AD (Table 1).

### AD mortality risk factors

50% of the population developed at least one cardiovascular event AD (Supplements, Table S1). Parameters that were associated significantly with survival were systolic BP, BMI (continuous and categorical), hemoglobin, albumin, and cardiovascular events (Table S1).

Multivariate cox analyses of AD parameters associated with AD mortality are shown in Table 2, identifying higher albumin, age and BMI AD as significant predictors (Table 2) while AD hemoglobin was no longer significant (Table 2).

**Table 2:** Multivariate Cox-analysis for the association between parameters AD and mortality^a^, Model1

Parameter	B	P-value	HR	95.0% CI	
				Lower	Upper
Age (years)	0.021	<0.001	1.021	1.009	1.033
BMI AD kg/m^2^ (reference = 18.5–24.9)					
25–29.9	−0.264	0.014	0.768	0.569	1.036
30–34.9	−0.559	0.084	0.572	0.409	0.800
35–39.9	−0.544	0.001	0.581	0.373	0.905
>40	−0.385	0.016	0.681	0.361	1.283
Albumin AD	−0.840	0.000	0.432	0.331	0.563

^a^The analysis, based on Cox regression, was adjusted for variables with significance of PV < 0.1 in the univariate analysis including all above covariates and AD hemoglobin, systolic blood pressures and type of renal replacement therapy (RRT). Variables were chosen using the forward-LR stepwise regression procedure with PV < 0.05 entrance criteria and PV < 0.1 for removal and then a cox-regression model was created.

Abbreviations: HR: hazard ratio, CI: confidence interval, BMI: body mass index; AD:6 month after dialysis; HF: heart failure.

### BD risk factors

**Figure 2:**
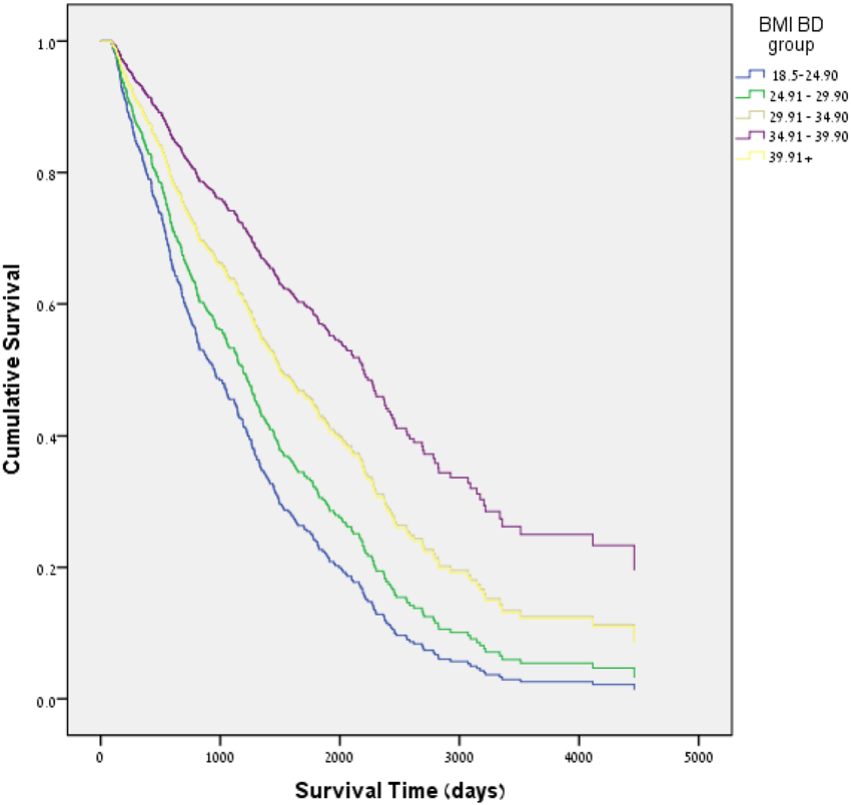
Cox regression survival plot indicating after dialysis (AD) cumulative survival for patients with diabetes and CKD by body mass index (BMI) group in Model 2. Patients in the BMI groups above 25 had improved AD survival compared with those of BMI 18.5–25.

Higher BMI, higher diastolic blood pressure (BP), pulse pressure (PP) of 40–60 versus PP < 40 and Hemoglobin >13 were significantly associated with longer survival (Table S2). Further categorization of BMI into sub-groups showed that compared to a normal BMI (18.5–25), a BMI of 35–40 (obesity grade 2) was associated with the best survival (HR 0.36 ± 0.23–0.55) (Fig. 2). A diastolic BP above 100 deemed protective compared to a diastolic BP < 80. There was no significant association between 1-year BD HbA1c, proteinuria and albuminuria and AD survival (Table S2).

Table 3 shows the adjusted hazard ratios of constant variables and BD variables on AD all-cause mortality. Age, BMI, PP and HF BD continued to be significant. Of note, BD diastolic BP, lipid levels and HbA1c were not significantly associated with mortality in the multivariate model.

**Table 3:** Adjusted Cox-analysis for the association between parameters one year BD to AD mortality in Model 2*.

Parameter	B	P-value	HR	95.0% CI	
				Lower	Upper
Age (years)	0.033	0.000	1.033	1.021	1.045
BMI BD kg/m^2^ (reference = 18.5–24.9)					
25–29.9	−0.388	0.014	0.678	0.497	0.925
30–34.9	−0.645	0.000	0.525	0.376	0.731
35–39.9	−0.968	0.000	0.380	0.245	0.588
>40	−0.563	0.027	0.570	0.346	0.937
HF BD**	0.427	0.000	1.533	1.228	1.915
Pulse pressure mmHG (reference = 40–60)					
<40	1.094	0.000	2.987	1.872	4.768
>60	0.296	0.015	1.344	1.059	1.706

*The analysis, based on Cox regression, was adjusted for Variables with Significance of PV < 0.1 in the univariate analysis including all above covariates and diabetes duration, socioeconomic status, hemoglobin, diastolic blood pressure, dialysis modality type, triglycerides and vascular comorbidity. Variables for the optimal model were selected using the forward-LR stepwise regression procedure with PV < 0.05 entrance criteria and PV < 0.1 for removal and a Cox-regression model was created.

**Cardiovascular comorbidity BD was significant but showed a high collinearity with HF. Because HF resulted in a better model it was included in this model.

Abbreviations: HR: hazard ratio, CI: confidence interval, BMI: body mass index; HF: heart failure; BD: before dialysis.

### Combined BD and AD analysis

A combined multivariate cox model of both BD and AD parameters (Table 4) that were independently and significantly associated with survival (PV < 0.1) was created using forward LR. Of the AD parameters only AD albumin continued to be significant. The BD parameters found to be significant after adjusting for AD parameters were age, PP < 40 or >60, BMI and cardiovascular comorbidity BD (including HF). Adjusted Cox Survival plots for the association between BMI BD or PP BD with AD mortality by model 3 are shown in Figures S1, S2, respectively (Supplements).

**Table 4:** A combined survival model for the association between both BD and AD parameters with AD mortality, Model 3A*.

Parameter	B	P-value	HR	95.0% CI	
				Lower	Upper
Age (years)	0.031	0.000	1.032	1.019	1.045
Albumin AD	−0.852	0.000	0.427	0.333	0.547
BMI BD kg/m^2^ (reference= 18.5–24.9)					
25–29.9	−0.290	0.103	0.749	0.529	1.060
30–34.9	−0.612	0.001	0.542	0.375	0.783
35–39.9	−0.802	0.001	0.448	0.281	0.715
>40	−0.550	0.051	0.577	0.333	1.002
Cardiovascular Comorbidity BD**	0.365	0.007	1.440	1.107	1.873
Pulse pressure mmHG (reference = 40–60)					
<40	1.091	0.000	2.978	1.741	5.095
>60	0.305	0.021	1.357	1.046	1.760

*Model 3A, based on multivariate Cox analysis, integrated BD and AD parameters that had been independently significantly associated with AD survival (PV < 0.1) in the univariate analysis for each time period. Variables for the optimal model were selected using the forward-LR stepwise regression procedure with PV < 0.05 entrance criteria and PV < 0.1 for removal and a Cox-regression model was created.

Abbreviations: HR: hazard ratio, CI: confidence interval, BMI: body mass index; HF: heart failure; AD: after dialysis; BD: before dialysis.

**Including: HF, CAD, PVD, stroke.

Additional models adjusting for cardiovascular AD comorbidity (Table S3) and AD HF (Table S4) continued to show significant association between BD BMI and BD PP with survival.

## Discussion

In this study we evaluated the hypothesis that the high rates of mortality in MD patients are partially derived from BD risk factors. To this end we generated a collective multivariate model where both BD and AD parameters were compared for independent association with mortality AD. A combined regression model with parameters BD and AD found that in diabetic patients, BD PP, BD BMI, BD cardiovascular comorbidity and AD albumin independently predict AD mortality (Table 4), even after adjusting for AD HF (Table S4) and AD cardiovascular comorbidity (Table S3 supplement).

Consistent with many previous studies (7–9), AD hypoalbuminemia was independently found to be a strong predictor of mortality in a multivariate model (Tables 2 and 4). High AD BMI was also associated with lower all-cause mortality (Table 2) but not when evaluated together with BD factors (Table 4). Of note, while all levels of AD obesity had beneficial effect compared to normal BMI, the maximal benefit of high BMI was in the range of 35–40. Because this association found in the multivariate AD model became insignificant in the combined AD + BD model, we suggest that the obesity paradox in MD may in fact begin already in the BD period. BD Obesity was found in this study to be a strong protective factor for AD mortality (Table 3, Figs 2 and S1). The optimal benefit was obtained in BMI BD of 35–40 (obesity grade 2) compared to normal BMI (HR 0.36). The result remained significant both in the multivariate model and in the “combined model,” with adjustment for AD parameters (Tables 3,4 and Supplementary material Tables S3, S4). Because the protective effect of obesity remained significant after adjusting for AD albumin, a normal BMI does not appear to reflect malnutrition. Thus, BD BMI emerges as a strong predictor of AD mortality. However, BD obesity may accelerate progression to ESRD.

The “obesity MD paradox” has been shown in a number of large-scale epidemiological studies^22–25^ where normal-weight adults on MD are at increased risk of mortality compared with overweight or obese people, despite the high positive association between BMI and chronic diseases^26^.

This phenomenon has also been described in the general population of diabetic patients^26^. However, in stages 3–5 CKD, only class I obesity was associated with a lower risk of death while obesity class II or III were not associated with a protective effect^27^. Because stages 3–5 in the general CKD population are widely heterogenous, our findings focus on diabetic BD with CKD stage 4 (Table S2) and suggest that the protective effect of obesity on AD mortality may begin already in the BD period.

High AD systolic blood pressure (SBP) was found in our study to positively correlate with longer survival. SBP > 160 was beneficial versus SBP < 120 (HR 0.67) (Table S1 supplement). This result is supported by observational studies that have consistently shown that increased mortality on MD is associated with low, but not high blood pressure^28,29^. A possible explanation may involve the additional risk factors for cardiovascular and heart failure present in MD patients, thereby reducing the added risk contribution of high BP. Furthermore, low BP in MD patients may indicate HF and carries an additional risk of severe hypotension during HD^30,31^.

Several studies found that wide PP AD predicts mortality in MD patients^32–35^. However in our study only BD PP, but not AD PP predicted AD mortality. Of note, in our study blood pressure measurements were out of dialysis collected from clinic or hospital data. Out of dialysis blood pressure has been recommended as a better predictor of mortality in MD patients^36^.

A narrow PP below 40 mmHg was associated with increased mortality when compared to PP > 60 mmHg. A similar finding was reported in a general MD population but only AD and not evaluated for the BD PP effect^37^. Both increased AD arterial stiffness and increased AD PP are predictors of AD mortality. Both low BP as well as PP < 45 mmHg were described as risk factors for mortality in patients with HF in the general population^38,39^. Low pulse pressure is independently related to elevated natriuretic peptides and increased mortality in advanced chronic heart failure^40^.

In our study, a narrow BD PP was a strong predictor for AD mortality and remained significant after adjusting for HF and vascular comorbidity BD (Table 3), for AD parameters (Table 4) and also when adjusting for newly-diagnosed HF AD (Table S4).

BD PP showed a U-shaped curve where PP values lower than 40 and higher than 60 were independently associated with higher mortality. Between the two, BD PP < 40 was a stronger predictor of AD death than PP > 60 (supplement, Figure S2). While a low PP reflects a very low cardiac output, a wide PP is an indicator of arterial stiffness, thereby also predicting morbidity and mortality^33,41–44^. A surrogate marker of wide PP, pulse wave velocity (PWV) was also reported to best predict death in CKD and especially in diabetics^45^.

Our results should be interpreted in the context of several limitations. Being a retrospective study, we can only describe associations and not causality. The number of patients was relatively small and derived from a single center, limiting the ability to find parameters that have a significant but small influence effect. The frequency difference of the tests between patients may also have affected the results: The BD test values were taken from the latest test over 12 months from MD onset, but for patients with low frequency tests, test values were taken from an earlier time. To address this issue, we examined whether there was a correlation between the results of the tests and the time from the beginning of MD in which they were taken, and no significant or high correlation was observed. Another limitation was that clinical and laboratory parameters were derived at only two time points - a year before initiation of dialysis and half a year after. These time points were selected as they represent relatively stable periods. Analysis using the average of the laboratory tests a year before dialysis yielded similar results (data not shown). Exclusion of patients that survived less than 90 days before dialysis could potentially lead to immortal time bias. We validated that excluded patients due to early mortality were, as expected, older and had lower BMI. However, our main focus was long-term outcome of chronic dialysis patients because around the start of dialysis there is a peak in mortality due to acute cardiovascular and infectious etiologies that skew death rates to overestimation of mortality in truly chronic dialysis patients^46,47^. Since in our study we aimed to asses only parameters that correlated with long term survival in MD patients, we followed previous larger studies^3,48^ and excluded early mortality. Of note, as the dialysis patient population comprised both peritoneal and hemodialysis patients, treatment time and access type could not be compared.

Finally, we aimed to find long term risk factors but it may be that one year BD is insufficient and that other effects may have occurred earlier.

The strengths of our findings include agreement with previous studies in MD patients highlighting low albumin and low AD BP as mortality predictors. Second, the study focused on a very specific population - only diabetics who reached ESRD and started MD. Thus, the specificity of this population may facilitate uncovering trends that distinguish this subgroup. Third, this is the first work to the best of our knowledge that attempts to examine BD parameters with adjustment to AD enabling the comparison of BD parameters with AD parameters.

## Conclusions

In this study, we found that BD parameters predominate as predictors of AD mortality in diabetics who reached ESRD and started MD. These parameters were BMI, PP and cardiovascular comorbidity. Our findings support the notion of Lamiere *et al*. who already in 2009 postulated that MD “patient survival is largely determined by the cardiovascular health status at initiation of dialysis and that even the best dialysis modality will not reverse the cardiovascular damage that has accumulated in the predialysis stage^8^”. Thus, in our study, both wide and narrow BD PP, normal BD BMI and BD cardiovascular comorbidity were identified as independent predictors of AD mortality even after adjusting for AD parameters. Whether this hypothesis can partially account for some of the negative cardiovascular randomized controlled trials in MD remains to be seen.

## Supplementary information

Supplementary Dataset 1
