# Supplementary material for: Risk Factors Before Dialysis Predominate as Mortality Predictors in Diabetic Maintenance Dialysis patients
Source: Sci Rep. 2019 Jul 23;9:10633. doi: 10.1038/s41598-019-46919-w (PMC6650444; doi:10.1038/s41598-019-46919-w)
Supplement: Supplementary file 1 — Supplementary Dataset 1 [file 41598_2019_46919_MOESM1_ESM.docx]

Risk Factors Before Dialysis Predominate as Mortality Predictors in Diabetic Maintenance Dialysis patients

Noa Tsur, Idan Menashe, Yosef S Haviv

Table S1: Unadjusted Cox-regression for the association between after dialysis (AD) parameters to AD mortality

|  | | N (%) or Mean ± SD | B | p-value | HR | 95.0% CI | |
| --- | --- | --- | --- | --- | --- | --- | --- |
| BMI AD | | 29.58±6.16 | -0.035 | <0.001 | 0.966 | 0.948 | .984 |
| BMI AD groups | 18.5-24.9 | 132 (23.5%) | [reference] | | | | |
|  | 25.0 - 29.9 | 203 (36.2%) | -0.284 | 0.036 | 0.752 | 0.577 | .982 |
|  | 30.0- 34.9 | 133 (23.7%) | -0.528 | 0.001 | 0.590 | 0.436 | .798 |
|  | 35.0 - 39.9 | 60 (10.7%) | -0.609 | 0.002 | 0.544 | 0.368 | .803 |
|  | ≥40 | 33 (5.9%) | -0.553 | 0.028 | 0.575 | 0.351 | .943 |
| Systolic blood pressure AD | | 137±22 | -0.005 | 0.059 | 0.995 | 0.990 | 1.000 |
| Systolic blood pressure AD (groups) | ≤ 119 | 110 (17.8%) | [reference] | | | | |
|  | 120- 139 | 222(35.9%) | -0.373 | 0.009 | 0.688 | 0.521 | 0.909 |
|  | 140- 159 | 191 (30.9%) | -0.343 | 0.017 | 0.710 | 0.536 | 0.939 |
|  | ≥160 | 95 (15.4%) | -0.417 | 0.018 | 0.659 | 0.467 | 0.930 |
| Diastolic Blood pressure AD | | 72.87±12.2 | -0.005 | 0.219 | 0.995 | 0.986 | 1.003 |
| diastolic blood pressure AD (groups) | ≤79 | 435 (70.4%) | [reference] | | | | |
|  | 80- 89 | 129 (20.9%) | -0.038 | 0.759 | 0.962 | 0.753 | 1.230 |
|  | 90- 99 | 40 (6.5%) | -0.149 | 0.484 | 0.861 | 0.567 | 1.309 |
|  | ≥100.00 | 14 (2.3%) | -0.121 | 0.722 | 0.886 | 0.455 | 1.725 |

| Table S1-continued | | | N (%) or Mean ± SD | B | p-value | H.R. | 95% CI | |
| --- | --- | --- | --- | --- | --- | --- | --- | --- |
| Pulse pressure (PP) AD | | | 65.1±18.1 | -0.004 | 0.157 | 0.996 | 0.990 | 1.002 |
| Pulse pressure AD (groups) | | 40-60 | 65.11±18.09 | [reference] | | | | |
|  |  | ≤40 | 41(7.65%) | 0.125 | 0.539 | 1.133 | 0.761 | 1.686 |
|  |  | ≥60 | 286 (53.4%) | -0.106 | 0.359 | 0.899 | 0.717 | 1.128 |
| Phosphorus AD | | | 5.28±1.65 | 0.038 | 0.300 | 1.039 | 0.966 | 1.118 |
| Albumin AD | | | 3.59±0.51 | -0.823 | <0.001 | 0.439 | 0.358 | 0.539 |
| HbA1c AD | | | 7.203±1.74 | 0.045 | 0.157 | 1.046 | 0.983 | 1.113 |
| hbA1c AD (groups) | | <= 7.00 | 285 (53.6%) | [reference] | | | | |
|  |  | >7.01 | 247 (46.4%)) | 0.07 | 0.54 | 1.32 | 0.86 | 1.07 |
| Hemoglobin AD | | | 11.36±1.5 | -0.098 | 0.002 | 0.907 | 0.852 | 0.965 |
| Hemoglobin AD groups | 10.01 - 13.01 | | 425 (66.3%) | [reference] | | | | |
|  | <= 10.00 | | 129 (20.1%) | 0.199 | 0.098 | 1.220 | 0.964 | 1.545 |
|  | 13.02+ | | 87 (13.6%) | -0.267 | 0.080 | 0.766 | 0.568 | 1.032 |
| Triglycerides AD | | | 173.6±132.4 | -0.001 | 0.166 | 0.999 | 0.998 | 1.000 |
| LDL AD | | | 88.4±37.5 | -0.001 | 0.561 | 0.999 | 0.995 | 1.003 |
| HDL AD | | | 41.327±12.5 | -0.003 | 0.620 | 0.997 | 0.985 | 1.009 |
| Cholesterol AD | | | 159.4±51.1 | <0.001 | 0.699 | 1.0 | 0.998 | 1.002 |
| First renal replacement therapy (RRT) | hemodialysis | | 612 (94%) | [reference] | | | | |
|  | peritoneal | | 36 (5.5%) | 0.388 | 0.06 | 1.47 | 0.98 | 2.21 |
|  | Transplant | | 4 (0.6%) | -0.174 | 0.84 | 0.76 | 0.27 | 2.6 |
| HF event AD | | | 204 (31%) | 0.18 | 0.07 | 1.19 | 0.98 | 1.45 |
| MI event AD | | | 126(19.3%) | -0.026 | 0.81 | 0.97 | 0.77 | 1.21 |
| Stroke event AD | | | 55 (8.4%) | 0.08 | 0.68 | 1.08 | 0.79 | 1.47 |
| PVD event AD | | | 99 (15.2%) | -0.001 | 0.99 | 0.99 | 0.78 | 1.26 |
| Cardiovascular comorbidity AD (inc.: HF,MI,stroke or pvd) | | | 324(49.7%) | 0.269 | 0.006 | 1.3 | 1.07 | 1.58 |

Table S2: Unadjusted Cox-regression for AD-mortality according to risk factors one year before dialysis (BD).

|  | | | N (%) or Mean±SD | B | p-value | H.R | 95 % CI | |
| --- | --- | --- | --- | --- | --- | --- | --- | --- |
| eGFR mean of the year BD | | | 26.5±19.4 | -0.002 | 0.49 | 0.998 | 0.994 | 1.003 |
| Systolic blood pressure BD | | | 141.48±21.2 | -0.001 | 0.83 | .999 | .995 | 1.004 |
| Systolic blood pressure BD (groups) | | <120 | 60 (12.7%) | [Reference] | | | | |
|  |  | 120.00 - 139.00 | 21(45.2%) | -0.14 | 0.44 | 0.87 | 0.61 | 1.24 |
|  |  | 140.00 - 159.00 | 177 (37.4%) | -0.14 | 0.44 | 0.87 | 0.60 | 1.24 |
|  |  | 160.00+ | 22 (4.7%) | 0.22 | 0.47 | 1.245 | 0.69 | 2.251 |
| Diastolic BP BD | | | 75.24±10.78 | -0.01 | 0.006 | .987 | 0.98 | .996 |
| Diastolic blood pressure BD (groups) | | <= 79.00 | 343 (61.4%) | [Reference] | | | | |
|  |  | 80.00 - 89.00 | 149 (26.7%) | -0.13 | 0.28 | 0.88 | 0.69 | 1.11 |
|  |  | 90.00 - 99.00 | 53 (9.5%) | -0.31 | 0.11 | 0.74 | 0.51 | 1.08 |
|  |  | 100.00+ | 14 (2.5%) | -0.83 | 0.03 | 0.44 | 0.20 | .93 |
| Pulse pressure BD | | | 66.25±17.31 | 0.01 | 0.12 | 1.01 | 0.99 | 1.01 |
| Pulse pressure BD (groups) | | 40.1 - 60.00 | 213 (38.1%) | [Reference] | | | | |
|  |  | <= 40.00 | 31 (5.5%) | 0.59 | 0.01 | 1.8 | 1.17 | 2.78 |
|  |  | >60.10 | 315 (56.4%) | 0.24 | 0.038 | 1.27 | 1.01 | 1.59 |
| HbA1C BD | | | 7.64±1.78 | 0.02 | 0.6 | 1.015 | 0.96 | 1.07 |
| HbA1c BD (dic.) | | <= 7.00 | 263 (43.4%) | [reference] | | | | |
|  |  | >7.01 | 342 (56.5%) | -0.02 | 0.85 | 0.98 | 0.8 | 1.2 |
| albumin BD | | | 3.62±0.49 | -0.15 | 0.12 | 0.86 | 0.71 | 1.04 |
| phosphorus BD | | | 4.78±1.35 | -0.03 | 0.4 | 0.96 | 0.9 | 1.04 |
| cholesterol BD | | | 174.76±53.6 | 0 | 0.68 | 1 | 0.998 | 1.001 |
| triglycerides BD | | | 178.62±119 | -0.001 | 0.057 | 0.99 | 0.998 | 1 |
| LDL BD | | | 98.29±42.7 | 0 | 0.708 | 1 | 0.998 | 1.003 |
| HDL BD | | | 41.90±13.2 | 0.003 | 0.42 | 1.003 | 0.996 | 1.001 |
| hemoglobin BD | | | 11.53±1.6 | -0.03 | 0.32 | 0.97 | 0.9 | 1.03 |
| Hemoglobin BD | 10-13 | | 422 (67.3%) | [Reference] | | | | |
|  | <10 | | 105 (16.7%) | -0.1 | 0.45 | 0.9 | 0.69 | 1.18 |
|  | >13 | | 100 (15.9%) | -0.29 | .033 | 0.74 | 0.57 | 0.98 |

|  | | N (%) or Mean±SD | B | p-value | H.R | 95 % CI | |
| --- | --- | --- | --- | --- | --- | --- | --- |
| BMI BD (Kg/m2) | | 30.93±7.35 | -0.03 | <0.001 | 0.97 | 0.94 | 0.98 |
| BMI BD (groups) | 18.5-24.9 | 86 (15.4%) | [reference] | | | | |
|  | 25 - 29.9 | 193 (34.4%) | -0.28 | 0.07 | 0.76 | 0.56 | 1.03 |
|  | 30 - 34.9 | 154 (27.4%) | -0.62 | <0.001 | 0.54 | 0.39 | 0.75 |
|  | 35 - 39.9 | 71 (12.6%) | -1.02 | 0.003 | 0.36 | 0.24 | 0.55 |
|  | ≥40 | 58 (10.3%) | -0.6 | <0.001 | 0.55 | 0.37 | 0.82 |
| Albuminuria BD (groups) | normoalbuminuria | 49 (10.5%) | [reference] | | | | |
|  | microalbuminuria | 75 (16.0%) | -0.07 | 0.75 | 0.93 | 0.59 | 1.45 |
|  | macroalbuminuria | 344 (73.5%) | -0.25 | 0.2 | 0.78 | 0.54 | 1.13 |

| Parameter | | B | P-value | HR | 95.0% CI | |
| --- | --- | --- | --- | --- | --- | --- |
|  |  |  |  |  | Lower | Upper |
|  | Age (years) | 0.03 | <0.001 | 1.031 | 1.018 | 1.044 |
|  | BMI BD kg/m^2^ _(reference= 18.5-24.9)_ |  |  |  |  |  |
|  | 25-29.9 | -0.23 | 0.187 | 0.79 | 0.56 | 1.12 |
|  | 30-34.9 | -0.54 | 0.004 | 0.58 | 0.41 | 0.84 |
|  | 35-39.9 | -4.72 | 0.002 | 0.49 | 0.31 | 0.77 |
|  | >40 | -0.46 | 0.10 | 0.63 | 0.37 | 1.09 |
|  | Pulse pressure mmHG (_reference=40-60)_ |  |  |  |  |  |
|  | <40 | 1.04 | <0.001 | 2.84 | 1.65 | 4.88 |
|  | >60 | 0.3 | 0.02 | 1.35 | 1.04 | 1.75 |
|  | Albumin AD | -0.86 | <0.001 | 0.43 | 0.33 | .545 |
|  | Cardiovascular Comorbidity BD** | 0.35 | 0.01 | 1.42 | 1.09 | 1.85 |
|  | Cardiovascular Comorbidity- AD** | 0.21 | 0.11 | 1.23 | 0.96 | 1.59 |

Table S3: Multivariate Cox regression Survival analysis for the association between parameters BD and AD to AD mortality, with adjustment for AD Cardiovascular comorbidity (Model 3C)

**including: HF, CAD, PVD, stroke

Table S4: Association between BD and AD parameters to AD mortality with adjustment to AD heart failure in Model 3B

| Parameter | | B | P-value | HR | 95.0% CI | |
| --- | --- | --- | --- | --- | --- | --- |
|  |  |  |  |  | Lower | Upper |
|  | Age (years) | 0.03 | <0.001 | 1.03 | 1.02 | 1.04 |
|  | BMI BD kg/m2 (reference= 18.5-24.9) |  |  |  |  |  |
|  | 25-29.9 | -0.21 | 0.23 | 0.81 | 0.57 | 1.14 |
|  | 30-34.9 | -0.53 | 0.004 | 0.59 | 0.41 | 0.89 |
|  | 35-39.9 | -0.72 | 0.002 | 0.49 | 0.31 | 0.78 |
|  | >40 | -0.47 | 0.09 | 0.63 | 0.36 | 1.08 |
|  | Pulse pressure mmHG (reference=40-60) |  |  |  |  |  |
|  | <40 | 1.09 | <0.001 | 2.98 | 1.74 | 5.09 |
|  | >60 | 0.3 | 0.02 | 1.36 | 1.05 | 1.76 |
|  | Albumin AD | -0.73 | <0.001 | 0.48 | 0.36 | 0.64 |
|  | Cardiovascular Comorbidity BD** | 0.38 | 0.01 | 1.46 | 1.12 | 1.91 |
|  | HF- AD | 0.05 | 0.73 | 1.05 | 0.81 | 1.36 |

Model 3A with additional adjustment to Heart failure AD.

*Model 3B, based on multivariate Cox analysis, combined BD and AD parameters that had been independently significantly associated with AD survival (PV<0.1) in the univariate analysis for each time period. The model was created using forward-LR stepwise regression procedure with PV<0.05 entrance criteria and PV<0.1 for removal.

Abbreviations: HR: hazard ratio, CI: confidence interval, BMI: body mass index; HF: heart failure; AD: after dialysis; BD: before dialysis.

**Including: HF, CAD, PVD, stroke.

Table S5: Adjusted cox analysis for the association between parameters before dialysis in diabetic CKD patients and the risk for transplantation.

(parameters as in model 2* )

| Parameter | | B | P-value | HR | 95.0% CI | |
| --- | --- | --- | --- | --- | --- | --- |
|  |  |  |  |  | Lower | Upper |
|  | Age (years) | -.133 | .000 | .875 | .831 | .922 |
|  | BMI BD kg/m^2^ _(reference= 18.5-24.9)_ |  |  |  |  |  |
|  | 25-29.9 | 10.862 | .915 | 52149.120 | .000 | 6.996E+09 |
|  | 30-34.9 | 10.548 | .917 | 38082.960 | .000 | 5.110E+09 |
|  | 35-39.9 | 10.386 | .918 | 32387.887 | .000 | 4.354E+09 |
|  | >40 | .149 | .999 | 1.161 | .000 | 5.764E+15 |
|  | HF BD | -1.141 | .073 | .320 | .092 | 1.111 |
|  | Pulse pressure BD (reference=40-60) |  |  |  |  |  |
|  | <40 | -10.701 | .935 | .000 | .000 | 2.008E+11 |
|  | >60 | .664 | .221 | 1.942 | .672 | 5.618 |

*Model 2 included parameters BD .

Parameters as in model 3A

| Parameter | | B | P-value | HR | 95.0% CI | |
| --- | --- | --- | --- | --- | --- | --- |
|  |  |  |  |  | Lower | Upper |
|  | Age (years) | -.139 | .000 | .871 | .821 | .923 |
|  | BMI BD kg/m2 (reference= 18.5-24.9) |  |  |  |  |  |
|  | 25-29.9 | 10.885 | .910 | 53348.086 | .000 | 2.180E+08 |
|  | 30-34.9 | 10.417 | .913 | 33439.310 | .000 | 1.366E+08 |
|  | 35-39.9 | 10.427 | .913 | 33750.680 | .000 | 1.382E+08 |
|  | >40 | .360 | .998 | 1.433 | .000 | 9.584E+15 |
|  | Cardiovascular comorbidity BD | -1.094 | .045 | .335 | .115 | .978 |
|  | Pulse pressure BD (reference=40-60) |  |  |  |  |  |
|  | <40 | -11.037 | .948 | .000 | .000 | 2.420E+13 |
|  | >60 | .478 | .394 | 1.613 | .538 | 4.841 |
|  | Albumin AD | 1.542 | .032 | 4.672 | 1.139 | 19.173 |

* Model 3A included parameters BD and AD .

Abbreviations: HR: hazard ratio, CI: confidence interval, BMI: body mass index; HF: heart failure; AD: after dialysis; BD: before dialysis.

Figure S1: Adjusted survival function by BMI BD group, by model 3A


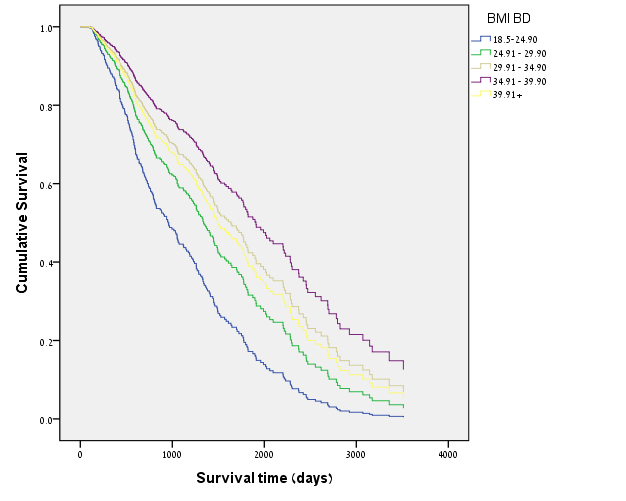


Figure S2: Adjusted Survival Function by Pulse pressure: by model 3A
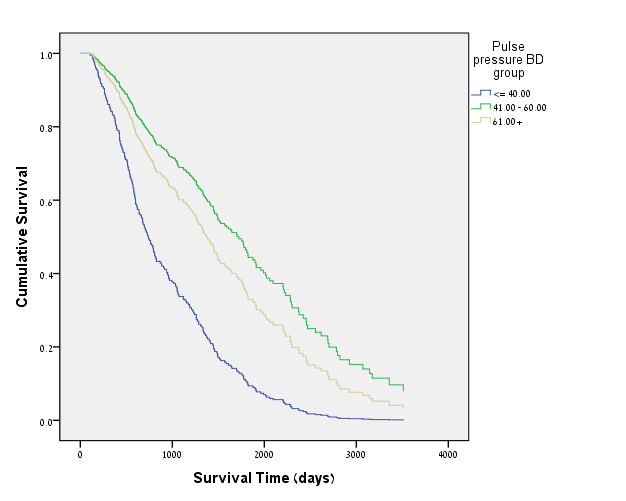


**Figure S3-S7: Graph showing proportionality assumption for the main variables**.

Figure S3

Cox regression survival plot for survival after dialysis of patients with diabetes and CKD by body mass index (BMI) group a year before dialysis. Patients in the BMI groups above 25 had improved AD survival compared with those of BMI 18.5-25.


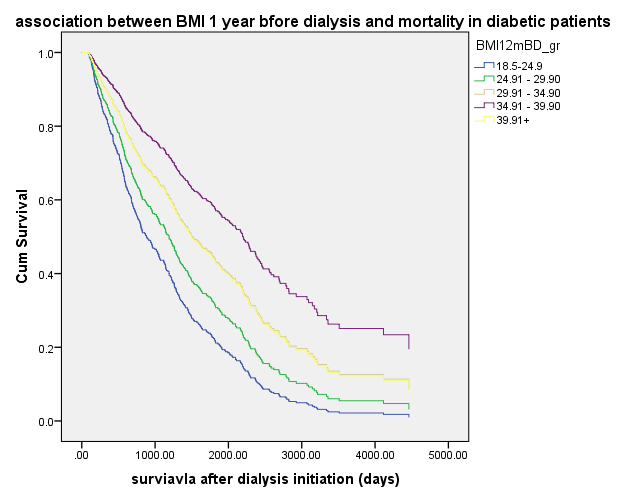


**Survival time after dialysis (days)**

Figure S4


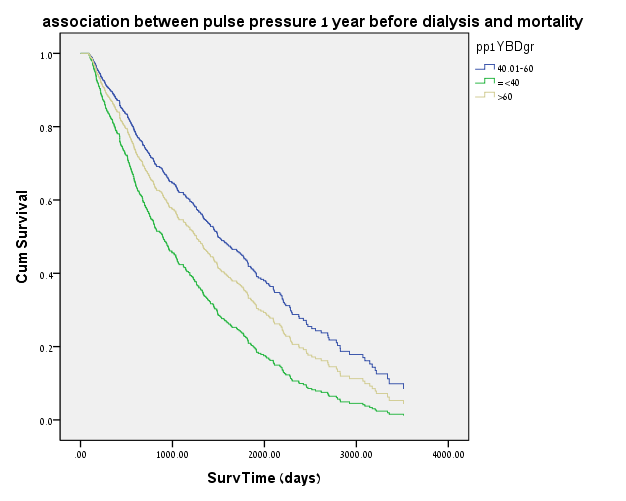


Cox regression survival plot for survival after dialysis of patients with diabetes and CKD by pulse pressure (PP) group a year before dialysis. Patients with PP between 40-60 had improved AD survival compared with those with PP >60 or PP<40.

Figure S5Figure S6


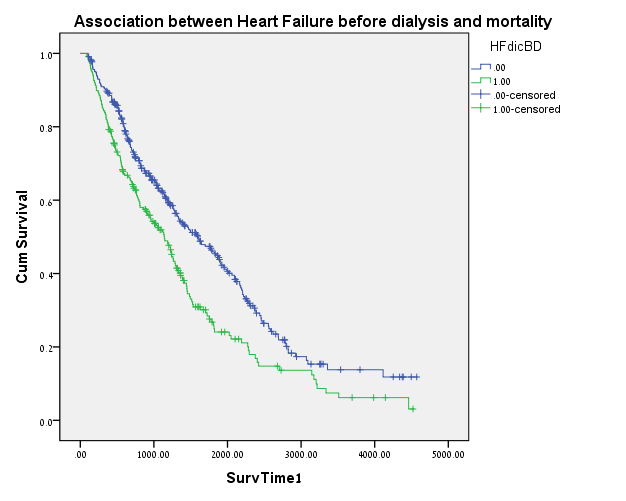


Cox regression survival plot for survival after dialysis of patients with diabetes and CKD by heart failure (HF) diagnosis begore dialysis. Patients with HF diagnosis before dialysis had poor survival compared with those without HF.


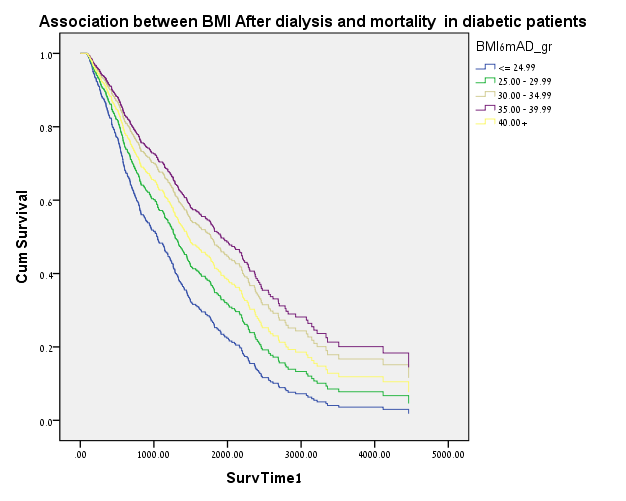


Cox regression survival plot for survival after dialysis of patients with diabetes and CKD by BMI group after dialysis (AD). Patients with BMI>25 had improved AD survival compared with those of BMI 18.5-25.

Figure S7


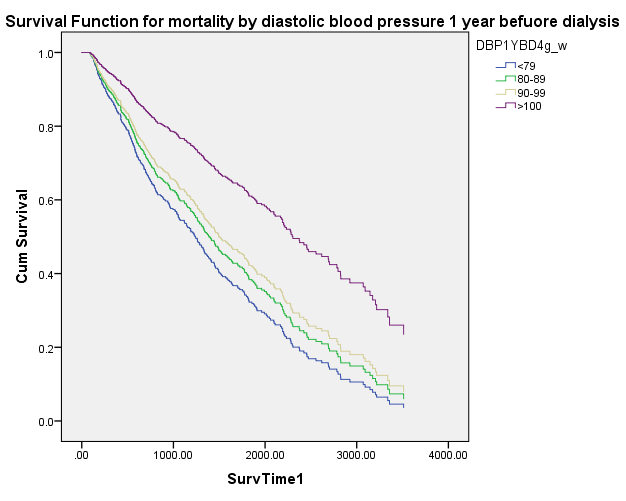


Cox regression survival plot for survival after dialysis (AD) of patients with diabetes and CKD by Diastolic blood pressure (DBP) group a year before dialysis. Patients with DBP >100 had improved AD survival compared with those DBP<79.
